# Resposta Exagerada da Pressão Arterial Sistólica ao Exercício e Isquemia Miocárdica à Ecocardiografia sob Estresse Físico

**DOI:** 10.36660/abc.20230047

**Published:** 2023-12-06

**Authors:** Cláudia Bispo Martins-Santos, Lara Teles Alencar Duarte, Cleovaldo Ribeiro Ferreira-Junior, Allexa Gabriele Teixeira Feitosa, Edvaldo Victor Gois Oliveira, Iana Carine Machado Bispo Campos, Enaldo Vieira de Melo, Stephanie Macedo Andrade, Antônio Carlos Sobral Sousa, Joselina Luzia Menezes Oliveira

**Affiliations:** 1 Universidade Federal de Sergipe São Cristóvão SE Brasil Universidade Federal de Sergipe , São Cristóvão , SE – Brasil; 2 Rede D’Or São Luiz Clínica e Hospital São Lucas Aracaju SE Brasil Rede D’Or São Luiz – Clínica e Hospital São Lucas , Aracaju , SE – Brasil; 3 Rede Primavera Setor de Métodos Gráficos Hospital Primavera Aracaju SE Brasil Rede Primavera – Setor de Métodos Gráficos do Hospital Primavera , Aracaju , SE – Brasil; 4 Fundação de Beneficência Hospital de Cirurgia Setor de Métodos Gráficos Aracaju SE Brasil Fundação de Beneficência Hospital de Cirurgia – Setor de Métodos Gráficos , Aracaju , SE – Brasil

**Keywords:** Pressão Arterial, Ecocardiografia sob Estresse, Teste de Esforço, Doença da Artéria Coronariana, Isquemia Miocárdica

## Abstract

**Fundamento:**

A associação entre resposta exagerada da pressão arterial sistólica ao exercício (REPASE) e isquemia miocárdica é controversa e pouco estudada em indivíduos com síndrome coronariana crônica estabelecida ou suspeita.

**Objetivo:**

Verificar a relação entre isquemia miocárdica e REPASE em indivíduos submetidos à ecocardiografia sob estresse físico (EEF).

**Métodos:**

Trata-se de estudo transversal com 14.367 indivíduos submetidos à EEF, de janeiro de 2000 a janeiro de 2022, divididos em dois grupos: G1 – composto por pacientes cuja pressão sistólica de pico apresentou incremento ≥ 90 mmHg (valor correspondente ao percentil 95 da população estudada) –, e G2 – formado por indivíduos que não apresentaram resposta hipertensiva exagerada. Os grupos foram comparados mediante os testes *t* de *Student* e qui-quadrado. Foram considerados significativos os valores de p < 0,05. Realizou-se, também, regressão logística para identificação de fatores de risco independentes para isquemia miocárdica, REPASE, queixa de precordialgia típica prévia ao exame e angina durante o teste.

**Resultados:**

Dos 14.367 pacientes, 1.500 (10,4%) desenvolveram REPASE e 7.471 (52,0%) eram do sexo feminino. Os percentuais de queixa prévia de precordialgia típica, angina durante o teste e isquemia miocárdica dos pacientes com REPASE foram de 5,8%, 2,4% e 18,1% contra 7,4%, 3,9% e 24,2%, em indivíduos sem REPASE, respectivamente (p = 0,021, p = 0,004, p < 0,001). Na análise multivariada, a REPASE foi associada, independentemente, a uma menor probabilidade de isquemia miocárdica (odds ratio: 0,73; intervalo de confiança de 95%: 0,58 a 0,93; p = 0,009).

**Conclusão:**

O incremento exagerado da pressão arterial sistólica durante a EEF pode ser um marcador de exclusão de isquemia miocárdica.

## Graphical abstract

**Figura Central:**
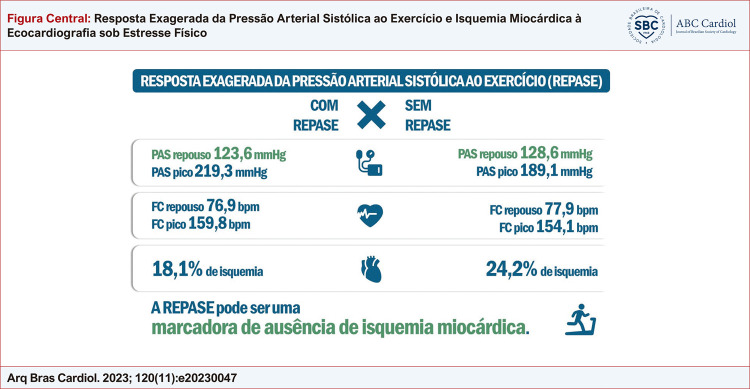
: Resposta Exagerada da Pressão Arterial Sistólica ao Exercício e Isquemia Miocárdica à Ecocardiografia sob Estresse Físico

## Introdução

A ecocardiografia sob estresse físico (EEF) consiste em prova reconhecida para avaliação da evolução funcional de coronariopatias. ^1 , 2^ Tal técnica permite acesso a diversos elementos da cascata isquêmica como angina pectoris, alterações eletrocardiográficas, além de mudanças da contratilidade segmentar e da função diastólica do ventrículo esquerdo (VE). ^1 , 3 - 6^

Tem sido sugerida possível associação entre a resposta exagerada da pressão arterial sistólica ao exercício (REPASE) e a presença de isquemia miocárdica. ^7 , 8^ Especula-se que a elevação intensa da pressão arterial durante o exercício causaria aumento do consumo miocárdico de oxigênio e, por conseguinte, isquemia subendocárdica, até mesmo na ausência de estenoses coronarianas significativas. ^9 - 11^ Assim sendo, a REPASE poderia estar associada ao aumento da ocorrência de eventos cardiovasculares, independentemente da capacidade cardiorrespiratória (CCR). ^12^

Em contrapartida, especula-se, também, que a resposta excessiva da pressão arterial sistólica (PAS) pode ser provocada pelo aumento do débito cardíaco (DC), consistindo, portanto, em resposta fisiológica que se traduz em prognóstico favorável, caracterizado por menor probabilidade de dor torácica e de isquemia miocárdica. ^13^ Desse modo, o incremento exagerado da PAS durante o exercício poderia ser um marcador de ausência de isquemia miocárdica. ^14 , 15^ A diminuição da CCR seria determinante para o aumento da mortalidade. ^16^

O objetivo da presente investigação foi verificar a frequência de isquemia miocárdica em pacientes com síndrome coronariana crônica (SCC) suspeita ou estabelecida que apresentaram ou não REPASE submetidos à EEF, bem como comparar suas diferenças clínicas e ecocardiográficas.

## Métodos

### Pacientes

Trata-se de estudo transversal a partir de base de dados construída prospectivamente, a qual compreende 14.503 indivíduos submetidos à EEF entre janeiro de 2000 e janeiro de 2022 no Laboratório de Ecocardiografia da Clínica e Hospital São Lucas (ECOLAB), referência cardiológica de Aracaju-Sergipe. Foram incluídos indivíduos acima de 18 anos encaminhados ao serviço, exceto aqueles que se recusaram a participar do estudo. Pacientes que tivessem usado betabloqueador até três dias antes do exame e os que não apresentaram aumento da PAS acima do seu respectivo valor basal com o exercício físico, também foram excluídos. Restando, portanto, 14.367 pacientes com SCC suspeita ou estabelecida.

Os pacientes foram divididos de acordo com a presença de REPASE, a qual foi definida por um aumento ≥ 90 mmHg (valor correspondente ao percentil 95 da população estudada). Desse modo, constituíram-se dois grupos: G1 – composto por 1.500 (10,4%) pacientes cuja PAS de pico apresentou incremento ≥ 90 mmHg –, e G2 – formado por 12.867 (89,6%) indivíduos que não exibiram resposta hipertensiva exagerada.

### Características clínicas

Os dados clínicos foram colhidos por meio de entrevistas realizadas antes do exame. Foi utilizado um questionário padronizado onde foram registrados: a ocorrência de sintomas como dispneia e dor torácica – sendo esta considerada como típica quando dor retroesternal desencadeada por esforço ou estresse emocional e aliviada ao repouso ou pelo uso de nitratos e atípica quando presentes somente dois desses fatores. ^17^ Também foram pesquisadas as medicações usadas, a presença de fatores de risco para SCC e o histórico de cardiopatia familiar ou pessoal, além de dados referentes à síndrome coronariana (SC) prévia.

Índices de massa corpórea maiores que 30 kg/m ^2^ foram caracterizados como obesidade. ^18^ Definiu-se hipercolesterolemia com base nos antecedentes pessoais e no uso de agente antilipidêmico (estatinas e/ou fibratos). ^19^ Indivíduos que referiram realizar menos que 150 minutos de atividade física de moderada intensidade ou 75 minutos de vigorosa intensidade foram considerados sedentários. ^20^ Considerou-se hipertensão arterial sistêmica (HAS) quando os níveis pressóricos aferidos no membro superior, em repouso e condições ideais, eram PAS ≥ 140 mmHg e/ou diastólica ≥ 90 mmHg, repetidos e confirmados, ou quando o paciente estava em uso de medicação anti-hipertensiva. ^21^

O diabete melito foi definido pela presença de glicemia em jejum ≥ 126 mg/dL, glicemia 2 horas após sobrecarga com 75 g de glicose ≥ 200 mg/dL, hemoglobina glicada ≥ 6,5% ou glicemia ao acaso ≥ 200 mg/dL associada a sintomas clássicos de hiperglicemia ou pelo uso de insulina ou agentes hipoglicemiantes orais. ^22^

Definiu-se infarto do miocárdio antigo mediante história clínica e/ou pela presença de alterações sugestivas em exames complementares prévios, como eletrocardiograma (ECG), ecocardiograma e/ou cineangiocoronariografia. ^6^

As indicações isoladas ou combinadas para a EEF foram: avaliação de precordialgia; avaliação pré-operatória para cirurgia não cardíaca; teste ergométrico (TE) positivo para isquemia miocárdica em pacientes com baixo risco para SC; TE positivo em paciente com risco intermediário ou alto para SC; TE negativo para isquemia miocárdica em pacientes com risco intermediário ou alto para SC; surgimento de arritmia durante o TE; estratificação de SCC previamente estabelecida e estratificação de risco pós-SC aguda. ^3 , 23^ Foram considerados TE sugestivos de isquemia aqueles que apresentaram infradesnivelamentos do segmento ST na fase de exercício ou recuperação com: morfologia horizontal ou descendente > 1 mm, aferido no ponto J; morfologia ascendente > 1,5 mm, em indivíduos de risco moderado ou alto para SC; e > 2 mm em indivíduos de baixo risco para SC, aferido no ponto Y. ^24^ As classificações de risco para SC seguiram o que é preconizado em Knuuti et al. ^5^ Apesar de traçados elétricos ao repouso com a presença de bloqueio de ramo esquerdo ou de alterações do segmento ST representarem limitações ^25^ aos critérios elétricos mencionados, nestes casos os demais segmentos que não envolvessem tais achados eram analisados, haja vista a aplicabilidade da EEF enquanto preditora de SC nestes cenários. ^2 , 26^

### Ecocardiografia sob estresse físico

O protocolo experimental básico consistia na realização de ECG de doze derivações e de ecocardiograma de repouso após a avaliação clínica. Em seguida, realizava-se o esforço físico em esteira rolante e, imediatamente após (em 30 segundos a 1 minuto), procedia-se a aquisição das imagens ecocardiográficas no pós-esforço imediato por um período de 2 a 5 minutos em esquema de análise simultânea (“ *side by side* ”) com as imagens obtidas no repouso para avaliação comparativa da contratilidade segmentar do ventrículo esquerdo como preconizado pela Sociedade Americana de Ecocardiografia. ^2^ Todos os pacientes foram submetidos aos protocolos padrão de Bruce ou de Ellestad durante o TE. ^24^ Todos os pacientes eram encorajados a atingir pico máximo da frequência cardíaca (FC), estimada pela equação: 220 – idade; já a FC submáxima foi definida como 85% da FC máxima. ^24^ Motivos para interrupção do esforço antes do alcance da FC máxima incluíram o desenvolvimento de angina, síncope, fadiga e arritmias malignas ou a critério do paciente por fadiga muscular. O consumo de oxigênio no pico do exercício (VO _2_ max) foi obtido, indiretamente, através de cálculos metabólicos padronizados que estimaram a capacidade aeróbica em cada estágio dos protocolos supracitados. ^27^ O VO _2_ max predito de acordo com sexo, idade, índice de massa corporal e nível de atividade física foi determinado a partir das equações de Almeida et al. ^28^ para população brasileira. A carga foi expressa também em equivalentes metabólicos (MET), em que 1 MET corresponde a 3,5 mL/kg·min de VO _2_ inspirado, referente ao repouso. ^29^ Durante o esforço, os indivíduos foram continuamente monitorados com ECG. Foram denominadas alterações eletrocardiográficas isquêmicas ao exercício a ocorrência de infradesnivelamentos do segmento ST que atendessem aos mesmos critérios supracitados para TE sugestivos de isquemia miocárdica. ^24^

Os indivíduos foram observados antes, durante e após o estresse sob esforço físico, através de ECG de doze derivações, para verificação de possíveis complicações decorrentes dele, as quais foram classificadas segundo as definições de Geleijnse et al. ^30^ A presença de morte, infarto agudo do miocárdio, acidente vascular encefálico, ruptura cardíaca, fibrilação ventricular e assistolia cardíaca foram consideradas complicações maiores. As complicações menores foram definidas como bloqueio atrioventricular, espasmo coronário e arritmias ventriculares (taquicardia ventricular não sustentada e extrassístoles ventriculares) bem como as supraventriculares (fibrilação atrial ou *flutter* , taquicardia supraventricular não sustentada e extrassístoles supraventriculares). ^30^

Era recomendada a suspensão de drogas cronotrópicas negativas, como os betabloqueadores, pelo menos três dias antes da realização das provas, mantendo-se as demais drogas usuais do paciente. ^2^

Os exames foram realizados com equipamento Hewlett Packard/Phillips SONOS 5500 até o ano de 2012 e, em seguida, por meio de um ecocardiógrafo Phillips IE-33, observando-se os aspectos técnicos vigentes descritos pela Sociedade Americana de Ecocardiografia. ^31 - 33^ As imagens ecocardiográficas bidimensionais foram obtidas nas janelas paraesternais e apicais, durante o repouso e imediatamente após o esforço, com o paciente em decúbito lateral esquerdo e sob registro eletrocardiográfico simultâneo. A motilidade segmentar da parede do VE foi avaliada por ecocardiografista experiente, conforme preconizado pela Sociedade Americana de Ecocardiografia. ^2^ O espessamento parietal segmentar do VE foi avaliado quantitativamente tanto no repouso como após o esforço, contemplando-se a metodologia de 16 segmentos, graduado em: 1, normal; 2, hipocinético; 3, acinético e 4, discinético. O índice de escore da motilidade do VE (IEMVE) foi calculado no repouso e durante o exercício como a soma dos escores conferidos a cada um dos 16 segmentos dividida pelo número de segmentos avaliados no dado momento. O IEMVE igual a 1 corresponde à normalidade, de 1,1 a 1,7 representa disfunção intermediária e maior que 1,7, disfunção importante. ^2^ A diferença entre o IEMVE de esforço e de repouso é chamada de ΔIEMVE. O desenvolvimento de nova alteração na motilidade parietal ou piora de dissinergia existente (ΔIEMVE ≠ 0) foi considerado indicativo de isquemia miocárdica. ^31 - 33^ A função diastólica do VE foi avaliada e classificada segundo as preconizações vigentes da Sociedade Americana de Ecocardiografia. ^34 , 35^

### Análise estatística

As variáveis quantitativas foram descritas como média e desvio-padrão e, conforme o pressuposto de normalidade amostral de todas variáveis, avaliado pelo teste Kolmogorov-Smirnov, elas foram analisadas por meio do teste t de Student para grupos independentes. Já as variáveis categóricas foram expostas em frequência absoluta e porcentagem. Para comparar as características das variáveis categóricas entre os dois grupos, utilizou-se o teste do qui-quadrado. Em todas as análises, a significância de 5% foi adotada. Para avaliação da associação entre os desfechos (REPASE, isquemia miocárdica e angina à EEF e queixa prévia de precordialgia típica) e os fatores associados, foram realizadas regressões logísticas pelo método hierárquico. Para entrar no modelo inicial, admitiram-se todas aquelas com p < 0,25, enquanto que, para permanecer no modelo de análise multivariada, adotou-se p < 0,05. As variáveis foram adicionadas e retiradas do modelo de forma manual, conforme critério citado anteriormente. As análises estatísticas foram processadas por meio do programa Statistical Package for the Social Sciences (SPSS), versão 22.0 (Chicago, IL, EUA). ^36^

### Aspectos éticos

Os princípios éticos que regem a experimentação humana foram cuidadosamente seguidos, e todos os pacientes assinaram termo de consentimento livre e esclarecido. O estudo foi aprovado pelo Comitê de Ética em Pesquisa da Universidade Federal de Sergipe (CAAE 1818.0.000.107-06).

## Resultados

### Características basais

A amostra foi composta por 14.367 indivíduos, com idade média de 58 ± 11 anos, sendo 52,0% de mulheres. Um total de 1.500 (10,4%) pacientes desenvolveram REPASE ( Tabela 1 ). A proporção de homens no grupo que apresentou incremento exagerado na PAS foi maior que entre aqueles que não desenvolveram REPASE. Em média, indivíduos com REPASE foram mais jovens do que os sem resposta excessiva. A principal indicação clínica para a EEF foi a avaliação de precordialgia, presente em 52,6% dos indivíduos. Em alguns casos, havia mais de uma indicação para o exame.

**Tabela 1:** – Características clínicas dos pacientes que apresentaram ou não resposta exagerada da pressão arterial sistólica ao exercício (REPASE)

Variáveis	REPASE n=1500 (10,4%)	Sem REPASE n=12867 (89,6%)	p ^*^
Sexo masculino	1008(67,2%)	5888(45,8%)	<0,001
Idade (média em anos)	54,6±10,3	58,1±11,4	<0,001
Hipertensão arterial sistêmica	972(65,5%)	7459(58,7%)	<0,001
Diabete melito	202(13,6%)	1651(13,0%)	0,516
Dislipidemia	819(55,2%)	7353(57,9%)	0,041
Fumantes	88(5,9%)	631(5,0%)	0,110
Sedentarismo	602(53,2%)	4783(52,7%)	0,726
Obesidade	489(32,8%)	2859(22,3%)	<0,001
Antecedentes familiares de SCC	831(56,0%)	7283(57,4%)	0,323
Antecedente pessoal de SCC	188(19,7%)	1738(21,4%)	0,242
Tratamento clínico para SCC ^†^	83(44,1%)	795(45,7%)	0,677
Angioplastia + *stent* ^†^	78(41,5%)	680(39,1%)	0,529
Revascularização cirúrgica ^†^	32(17,5%)	344(20,1%)	0,397
Infarto prévio antigo (> 60 dias)	76(5,3%)	680(5,5%)	0,730
Infarto prévio recente (< 60 dias)	0(0,0%)	48(0,4%)	0,018
**Sintomatologia ^**‡**^**			
– Assintomáticos	648(45,0%)	5209(42,0%)	0,028
– Precordialgia típica prévia ao exame	83(5,8%)	922(7,4%)	0,021
– Precordialgia atípica prévia ao exame	644(44,7%)	5630(45,4%)	0,642
– Dispneia prévia ao exame	69(4,8%)	731(5,9%)	0,091
Bloqueio de ramo esquerdo	42(2,8%)	534(4,2%)	0,012
**Uso de anti-hipertensivos**	723(49,4%)	6020(47,8%)	0,271
– Diurético	17(3,7%)	200(4,1%)	0,657
– IECA	159(10,9%)	1159(9,2%)	0,040
– BRA II	346(23,7%)	2672(21,3%)	0,034
– Bloqueador de canal de cálcio	121(8,3%)	918(7,3%)	0,178
– Betabloqueador	303(20,7%)	2887(23,0%)	0,052
– Nitrato	40(2,7%)	357(2,8%)	0,814
**Indicações clínicas para a EEF ^**§**^**			
– Investigação de precordialgia	727(50,5%)	6552(52,8%)	0,097
– Pré-operatório de cirurgia não cardíaca	58(6,2%)	585(7,4%)	0,170
– TES em indivíduos com baixo risco para SC	221(23,6%)	1675(21,2%)	0,099
– TES em indivíduos com risco intermediário ou alto para SC	41(4,4%)	436(5,6%)	0,133
– TEN em indivíduos com risco intermediário ou alto para SC	12(1,3%)	112(1,4%)	0,730
– Arritmias durante TE	1(0,1%)	48(0,6%)	0,051
– Estratificação de risco de SCC	188(19,7%)	1738(21,4%)	0,242

BRA II: bloqueador do receptor da angiotensina II; EEF: ecocardiografia sob estresse físico; IECA: inibidor da enzima conversora da angiotensina; SC: síndromes coronárias; SCC: síndrome coronariana crônica; TE: teste ergométrico; TEN: teste ergométrico negativo para isquemia miocárdica; TES: teste ergométrico sugestivo de isquemia miocárdica. (*) As variáveis qualitativas foram calculadas por meio do método de qui-quadrado de Pearson, e, as variáveis quantitativas, por meio do teste t de Student para amostras independentes, conforme o pressuposto de normalidade da amostra. (†) Para a análise das terapêuticas de SCC (tratamento clínico, angioplastia + stent e revascularização cirúrgica), foram considerados exclusivamente os pacientes com coronariopatia (n = 1926). (‡) Para a análise da sintomatologia, foram considerados apenas os dados completos (n = 13936). (§) Para a análise das indicações clínicas, foram considerados apenas os dados completos (n = 12394). Fonte: dados coletados pelos autores.

Os pacientes com REPASE apresentaram frequência menor de dislipidemia e maior de HAS e de obesidade. Ademais, o grupo com REPASE usava mais inibidores da enzima conversora da angiotensina e de bloqueadores do receptor da angiotensina II. Quanto à sintomatologia, a maior parte de assintomáticos pertencia ao grupo REPASE, enquanto indivíduos sem incremento sistólico exagerado apresentaram maior frequência de queixa prévia de precordialgia típica que os com REPASE ( Tabela 1 ).

### Ecocardiografia sob estresse físico

**Figura 1:**
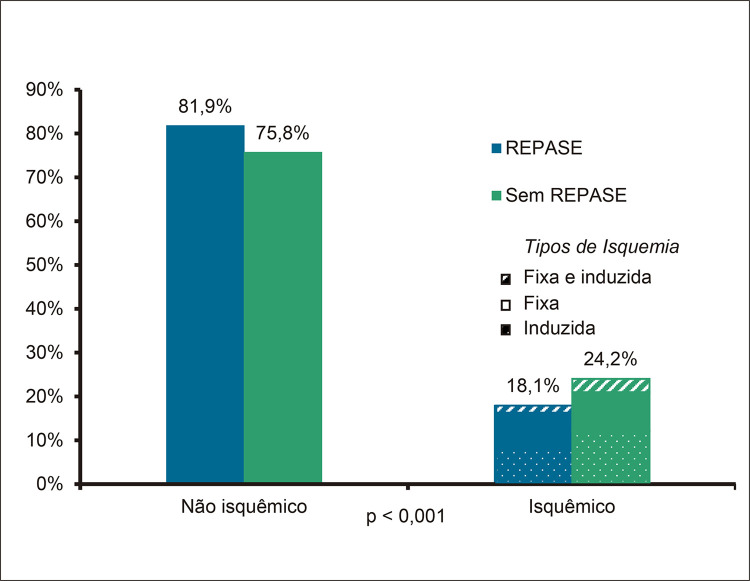
– Comparação da frequência de isquemia miocárdica à ecocardiografia sob estresse físico entre os grupos que apresentaram ou não resposta exagerada da pressão arterial sistólica ao exercício (REPASE). Fonte: dados coletados pelos autores.

Não houve registro de complicações maiores. A maioria dos pacientes que referiram angina durante o teste eram do grupo sem REPASE ( Tabela 2 ). Houve frequência maior de infradesnivelamento de ST no grupo com REPASE e de alterações ecocardiográficas compatíveis com presença de isquemia miocárdica nos indivíduos sem REPASE ( Figura 1 ), bem como de maiores níveis de IEMVE ao repouso e no esforço ( Tabela 2 ).

**Tabela 2:** – Características ecocardiográficas e ergométricas dos pacientes que apresentaram ou não resposta exagerada da pressão arterial sistólica ao exercício (REPASE)

Variáveis	REPASE n=1500 (10,4%)	Sem REPASE n=12867 (89,6%)	p ^*^
**Resultado da cinética segmentar ^**†**^**			
– Normal	1227(81,9%)	9751(75,8%)	<0,001
– Isquemia induzida	127(8,5%)	1563(12,2%)	<0,001
– Isquemia fixa	121(8,1%)	1151(9,0%)	<0,001
– Isquemia fixa e induzida	24(1,6%)	391(3,0%)	0,002
PAS no repouso (mmHg)	123,6±12,1	128,6±12,7	<0,001
PAS no pico (mmHg)	219,3±16,3	189,1±16,6	<0,001
PAS após esforço (mmHg)	156,5±25,8	145,0±20,7	<0,001
FC no repouso (bpm)	76,9±13,1	77,9±14,0	0,005
FC no pico (bpm)	159,8±16,8	154,1±19,2	<0,001
FC após esforço (bpm)	101,7±15,3	98,0±18,3	<0,001
DP no repouso (×10 ^3^ mmHg.bpm)	9,5±2,0	10,0±2,2	<0,001
DP no pico (×10 ^3^ mmHg.bpm)	35,0±4,0	29,2±4,7	<0,001
DP após esforço (×10 ^3^ mmHg.bpm)	15,9±3,7	14,2±3,5	<0,001
% da FC máxima teórica	96,7±8,3	95,2±9,7	<0,001
**Alcance da FC no teste ^**‡**^**			
– Abaixo da submáxima	119(7,9%)	1657(12,9%)	<0,001
– Submáxima	619(41,3%)	5138(40,0%)	0,329
– Máxima	211(14,1%)	1392(10,8%)	<0,001
– Acima da máxima	551(36,7%)	4670(36,3%)	0,754
Angina durante o teste	36(2,4%)	500(3,9%)	0,004
Dispneia durante o teste	164(11,0%)	1421(11,1%)	0,951
**Infradesnivelamento de ST**	883(59,3%)	6824(53,8%)	<0,001
– Ascendente ^§^	325(36,8%)	2661(39,0%)	0,209
– Retificado ^§^	318(36,0%)	2669(39,1%)	0,075
– Descendente ^§^	241(27,3%)	1499(22,0%)	<0,001
Bloqueio atrioventricular	3(0,9%)	18(0,5%)	0,304
Espasmo coronário	0(0,0%)	1(0,0%)	0,766
TV não sustentada	1(0,3%)	24(0,6%)	0,448
Extrassístoles ventriculares	75(21,6%)	1012(25,8%)	0,088
Fibrilação atrial ou *flutter*	0(0,0%)	8(0,2%)	0,399
TSV não sustentada	5(1,4%)	59(1,5%)	0,925
Extrassístoles supraventriculares	30(8,7%)	406(10,4%)	0,313
Tempo de esteira em minutos	8,2±2,6	7,1±2,6	<0,001
Capacidade funcional (METs)	10,5±3,1	9,7±3,0	<0,001
VO _2max_ predito (mL.kg ^-1.^ min ^-1^ )	26,3±6,0	24,5±6,2	<0,001
VO _2max_ atingido (mL.kg ^-1.^ min ^-1^ )	36,7±10,7	34,1±10,5	<0,001
Razão VO _2max_ atingido por predito (%)	143,2±38,9	143,2±42,8	0,986
Fração de ejeção do VE (%)	67,1±6,4	67,1±6,7	0,808
Índice de massa do VE (g/m ^2^ )	88,6±22,1	84,9±22,9	<0,001
Volume do átrio esquerdo (mL/m ^2^ )	28,8±13,1	29,3±14,2	0,400
IEMVE repouso	1,02±0,1	1,03±0,1	0,001
IEMVE ao esforço	1,03±0,1	1,04±0,1	<0,001
**Função diastólica ^**//**^**			
– Normal	230(20,4%)	1998(22,2%)	0,163
– Disfunção grau I	618(54,7%)	5016(55,7%)	0,531
– Disfunção grau II	277(24,5%)	1950(21,7%)	0,028
– Disfunção grau III	4(0,4%)	38(0,4%)	0,738

DP: duplo produto; FC: frequência cardíaca; IEMVE: índice de escore de motilidade do ventrículo esquerdo; PAS: pressão arterial sistólica; TSV: taquicardia supraventricular; TV: taquicardia ventricular; VE: ventrículo esquerdo; VO _2max_: consumo máximo de oxigênio. ( ^*^ ) As variáveis qualitativas foram calculadas por meio do método de qui-quadrado de Pearson, e, as variáveis quantitativas, por meio do teste t de Student para amostras independentes, conforme o pressuposto de normalidade da amostra. ( ^†^ ) Para a análise da cinética segmentar, foram considerados apenas os dados completos (n = 14355). ( ^‡^ ) Para a análise da FC alcançada, apenas os dados completos foram considerados (n = 14357). ( ^§^ ) Para a análise das morfologias do infradesnivelamento do segmento ST (ascendente, retificado e descendente), foram considerados exclusivamente os pacientes que apresentaram depressão do segmento (n = 7707). ( ^//^ ) Para a análise da função diastólica, foram considerados apenas os dados completos (n = 10131). Fonte: dados coletados pelos autores.

À análise multivariada, manteve-se uma relação inversa e significativa entre isquemia miocárdica e REPASE ( Tabela 3 ). O maior preditor de isquemia miocárdica foi o aparecimento de angina durante o teste ( Tabela 4 ). A REPASE não se associou à precordialgia típica prévia ao exame ( Tabela 5 ), tampouco ao surgimento de angina durante o teste ( Tabela 6 ).

**Tabela 3:** – Regressão logística multivariada com parâmetros associados à resposta exagerada da pressão arterial sistólica ao exercício à ecocardiografia sob estresse físico

Variáveis	*Odds ratio*	IC 95%	p
Isquemia miocárdica	0,72	0,59-0,87	0,001
Sexo masculino	2,38	2,05-2,77	<0,001
Idade	0,98	0,97-0,99	<0,001
Hipertensão arterial sistêmica	1,42	1,21-1,65	<0,001
Síndrome coronariana crônica	0,85	0,70-1,03	0,103
Obesidade	1,54	1,32-1,79	<0,001
Angina típica prévia ao exame	0,83	0,61-1,13	0,229
Dor típica ao exame	0,81	0,52-1,27	0,367

IC 95%: intervalo de confiança de 95%. Fonte: dados coletados pelos autores.

**Tabela 4:** – Regressão logística multivariada com parâmetros associados à presença de isquemia miocárdica à ecocardiografia sob estresse físico

Variáveis	*Odds ratio*	IC 95%	p
REPASE	0,73	0,58-0,93	0,009
Sexo masculino	1,33	1,15-1,55	<0,001
Idade	1,00	1,00-1,01	0,361
Hipertensão arterial sistêmica	1,10	0,94-1,28	0,241
Diabete melito	1,32	1,10-1,58	0,003
Dislipidemia	1,39	1,21-1,61	<0,001
Síndrome coronariana crônica	3,69	3,16-4,30	<0,001
Tabagismo	1,44	1,08-1,91	0,012
Antecedente familiar de SCC	1,38	1,19-1,59	<0,001
Angina típica prévia ao exame	2,38	1,87-3,02	<0,001
Angina durante o teste	11,03	7,60-16,01	<0,001
Infradesnivelamento de ST	1,53	1,33-1,76	<0,001
Capacidade funcional (METs)	0,94	0,91-0,97	<0,001
Fração de ejeção do VE (%)	0,94	0,93-0,95	<0,001

IC 95%: intervalo de confiança de 95%; REPASE: resposta exagerada da pressão arterial sistólica ao exercício; SCC: síndrome coronariana crônica; VE: ventrículo esquerdo. Fonte: dados coletados pelos autores

**Tabela 5:** – Regressão logística multivariada com parâmetros associados à presença de queixa de precordialgia típica prévia ao exame

Variáveis	*Odds ratio*	IC 95%	p
REPASE	0,80	0,56-1,14	0,218
Isquemia miocárdica ecocardiográfica	2,37	1,88-3,00	<0,001
Sexo masculino	1,05	0,84-1,31	0,676
Idade	0,99	0,98-0,995	0,003
Hipertensão arterial sistêmica	1,06	0,85-1,33	0,602
Diabete melito	1,10	0,84-1,44	0,501
Dislipidemia	1,32	1,06-1,64	0,013
Síndrome coronariana crônica	1,01	0,78-1,30	0,964
Tabagismo	1,81	1,24-2,64	0,002
Angina durante o teste	6,52	4,73-8,99	<0,001
Infradesnivelamento de ST	0,89	0,72-1,09	0,256
Capacidade funcional (METs)	0,95	0,91-0,99	0,008

IC 95%: intervalo de confiança de 95%; REPASE: resposta exagerada da pressão arterial sistólica ao exercício. Fonte: dados coletados pelos autores.

**Tabela 6:** – Regressão logística multivariada com parâmetros associados ao aparecimento de angina durante a ecocardiografia sob estresse físico

Variáveis	*Odds ratio*	IC 95%	p
REPASE	1,12	0,68-1,85	0,651
Isquemia miocárdica	10,37	7,21-14,91	<0,001
Sexo masculino	1,63	1,17-2,26	0,004
Idade	0,98	0,96-0,99	0,003
Hipertensão arterial sistêmica	1,01	0,72-1,42	0,950
Diabete melito	1,04	0,71-1,51	0,856
Dislipidemia	0,89	0,65-1,22	0,471
Síndrome coronariana crônica	1,02	0,73-1,43	0,892
Angina típica prévia ao exame	6,51	4,72-8,97	<0,001
Infradesnivelamento de ST	1,70	1,25-2,32	0,001
Capacidade funcional (METs)	0,83	0,78-0,88	<0,001

IC 95%: intervalo de confiança de 95%; REPASE: resposta exagerada da pressão arterial sistólica ao exercício. Fonte: dados coletados pelos autores.

## Discussão

No presente estudo, a REPASE associou-se a menor probabilidade de queixa de precordialgia típica prévia, de angina induzida pelo exercício e de isquemia miocárdica à EEF, com menores IEMVE ao repouso e esforço. Contudo, o grupo que apresentou REPASE também se associou a maior presença de alterações eletrocardiográficas sugestivas de isquemia miocárdica.

A associação entre o infradesnivelamento do segmento ST e a REPASE observada nesta pesquisa pode ser explicada pela relação entre o incremento sistólico e os falsos positivos para isquemia miocárdica à eletrocardiografia, descrita em estudos prévios. ^37 , 38^ Possivelmente, outra teoria seria a presença de doença microvascular coronariana nos pacientes com infradesnivelamento de ST, ^39^ a qual ocasionaria funcionamento distinto da bomba cardíaca em comparação aos indivíduos com SC obstrutivas, o que não impediria o alcance de maiores níveis pressóricos no primeiro grupo. Daubert et al. ^40^ observaram que a combinação de ECG sugestivo de isquemia miocárdica com ecocardiograma normal ao esforço associou-se a maiores níveis pressóricos no pico da EEF.

Diferentemente de outros trabalhos, nossa amostra era composta em sua maioria por mulheres (52,0%), e, inobstante, houve manutenção da predileção da REPASE pelo sexo masculino, constatação também verificada na literatura, bem como a predominância de pacientes mais jovens com resposta sistólica exagerada ao exercício. ^41 - 44^ Não foram registradas complicações maiores no presente estudo. A EEF é uma modalidade com baixa prevalência de eventos adversos que representem risco de morte. ^45^

O presente estudo evidenciou maior alcance de METs entre aqueles com REPASE. Possivelmente, tal situação esteja atrelada ao fato do grupo REPASE apresentar indivíduos mais jovens e, por conseguinte, com maior facilidade de incremento do duplo produto, aumentando o trabalho cardíaco. ^42 , 44 , 46^ Kokkinos et al. ^16^ encontraram associação entre a baixa CCR e o aumento da mortalidade por todas as causas, sendo o risco substancialmente maior (47% versus 92%) entre aqueles que não conseguiram um incremento superior a 52 mmHg na PAS no pico do esforço. Tal descoberta corrobora com o conceito de que a resposta da PAS ao exercício fornece informações essenciais acerca da integridade do sistema cardiovascular. ^13^

O predomínio da HAS no grupo REPASE é esperado, o que proporciona alcance de maiores níveis pressóricos – padrão observado também em outros estudos. ^14 , 41 , 44^ Quanto aos demais fatores de risco cardiovascular, os indivíduos com incremento sistólico exagerado associaram-se também à obesidade no presente estudo. Giang et al. ^46^ demonstraram que pacientes com elevados níveis pressóricos ao exercício apresentavam maiores índices de massa corporal. Além disso, a dislipidemia foi mais prevalente entre os indivíduos sem REPASE em nossa amostra. Bouzas-Mosquera et al. ^41^ encontraram associação distinta, na qual a dislipidemia correlacionou-se ao exagero da resposta sistólica ao exercício.

Há relatos de que o uso de anti-hipertensivos não exerce influência significativa sobre a resposta sistólica exagerada ao exercício. ^14 , 41 - 44^ Contrariamente, nossa amostra exibiu associação entre a REPASE e o maior uso de inibidores da enzima conversora da angiotensina e de bloqueadores dos receptores da angiotensina II. Todavia, este achado pode ser justificado pela maioria de hipertensos neste grupo, uma vez que as duas drogas compõem a primeira linha no tratamento da HAS. ^21^

A CCR e o histórico prévio do indivíduo deverão ser ponderados durante a avaliação da REPASE, especialmente no que tange aos atletas. Em população de atletas, Caselli et al. ^47^ encontraram valores de corte para a REPASE acima de 220 mmHg para homens e de 200 mmHg para mulheres, e demonstraram ainda associação entre o incremento sistólico exagerado e os esportes de *endurance* (definidos enquanto atividades primariamente isotônicas) e as modalidades com componentes isotônicos e isométricos. Desse modo, atletas destas modalidades esportivas específicas poderiam apresentar REPASE sem, no entanto, associar-se a patologias como a HAS. ^47^

Optamos por definir a REPASE enquanto aumento na PAS maior ou igual ao percentil 95 da nossa população a fim de adequar o incremento às características intrínsecas da amostra, parâmetro também utilizado em outros estudos, ^42 - 44^ permitindo reprodutibilidade. Nessas pesquisas, conduzidas em população europeia, os deltas de incremento sistólico referentes ao percentil 95 de suas respectivas amostras foram valores menores que o de nossa população (≥ 80 e ≥ 70 mmHg). ^42 - 44^

Nossos resultados não invalidam o efeito benéfico oriundo da redução de ambas pressões sistólicas (em repouso e no pico do esforço) induzida pela atividade física regular. ^48^ Em nossa amostra, o valor médio do índice de massa do VE foi maior entre indivíduos com REPASE, o que pode ser oriundo do remodelamento do VE induzido pelos elevados níveis pressóricos. Perçuku et al., ^8^ baseados em metanálise com oito estudos longitudinais que incluíram um total de 47.188 pacientes sem doença arterial coronariana, concluíram que a REPASE constitui um fator de risco independente de eventos cardiovasculares e mortalidade. Para verificar se a relação inversa entre isquemia miocárdica e REPASE encontrada no presente estudo poderia indicar um fator de proteção independente para eventos cardiovasculares, análises futuras são necessárias.

Não houve associação entre história pessoal positiva para SCC e ausência de REPASE em nosso estudo, conjuntura também observada em estudos europeus. ^42 , 43^ Contudo, no presente trabalho, a REPASE manteve-se como um fator de proteção ao surgimento de isquemia miocárdica à análise multivariada. Outro trabalho que dispunha de dados de angiografia demonstrou menor frequência de doença coronariana em pacientes com REPASE. ^13^

Durante o estresse físico, espera-se, em indivíduo saudável, aumento progressivo da PAS concomitantemente ao incremento do trabalho cardíaco, enquanto a pressão arterial diastólica permanece constante ou discretamente reduzida. ^49^ O incremento da PAS resulta do aumento no DC, o qual, por sua vez, é proveniente das elevações da FC e do volume de ejeção. Simultaneamente, a resposta simpática redistribui o fluxo sanguíneo para áreas com maior necessidade metabólica, produzindo vasodilatação muscular e vasoconstrição em áreas inativas, o que explica a discreta redução da pressão arterial diastólica. ^49 , 50^ Embora um aumento do DC proporcione melhores prognósticos, um aumento inesperado da resistência vascular periférica em circunstâncias de estresse físico pode ser um indicador de pior prognóstico. ^49^

Tais diferenças entre os dois determinantes do incremento da PAS ao exercício – aumento do DC e aumento da resistência vascular periférica – podem explicar, em partes, as inconsistências apresentadas entre os diversos estudos acerca da presença de REPASE e o efeito na mortalidade por todas as causas. Consonantemente, Daubert et al. ^40^ observaram que indivíduos com isquemia miocárdica à EEF alcançavam menor duplo produto. Em nosso trabalho, os indivíduos com REPASE partiram de valores de PAS mais baixos e apresentaram maior aumento tanto da PAS quanto da FC com o exercício, o que corrobora com a hipótese de que o aumento do DC poderá ser o principal determinante para os presentes resultados.

### Limitações

Nosso estudo apresenta as limitações inerentes a estudos observacionais transversais cuja amostra provém de um único centro, assim sendo, destacamos que a compreensão da influência da REPASE na mortalidade e nos eventos cardiovasculares requer realização de estudos longitudinais que possam observar os desfechos nesta população. Salienta-se que os resultados são referentes a pacientes com SCC estabelecida ou suspeita. Ademais, apesar da exclusão de pacientes tratados com betabloqueadores três dias antes do teste, não se pode descartar um efeito residual dessas drogas. Reitera-se que indivíduos que não apresentaram incremento na PAS com o esforço acima do valor basal foram excluídos.

## Conclusões

O incremento exagerado da PAS durante a EEF pode ser um marcador associado à ausência de isquemia miocárdica em pacientes com SCC conhecida ou suspeita. Portanto, o uso da EEF expressa importante vantagem no que diz respeito à possibilidade de acessar a capacidade aeróbica do paciente e, com isso, compreender o comportamento da resposta pressórica sistólica ao exercício. Ademais, salienta-se que o valor prognóstico do incremento sistólico exagerado demandará a realização de novos estudos.
