# Supplementary material for: Resposta Exagerada da Pressão Arterial Sistólica ao Exercício e Isquemia Miocárdica à Ecocardiografia sob Estresse Físico
Source: Arq Bras Cardiol. 2023 Dec 6;120(11):e20230047. [Article in Portuguese] doi: 10.36660/abc.20230047 (PMC10773463; doi:10.36660/abc.20230047)
Supplement: Translation

# Translation: Exaggerated Systolic Blood Pressure Increase with Exercise and Myocardial Ischemia on Exercise Stress Echocardiography

**Authors:** Cláudia Bispo Martins-Santos, Lara Teles Alencar Duarte, Cleovaldo Ribeiro Ferreira-Junior, Allexa Gabriele Teixeira Feitosa, Edvaldo Victor Gois Oliveira, Iana Carine Machado Bispo Campos, Enaldo Vieira de Melo, Stephanie Macedo Andrade, Antônio Carlos Sobral Sousa, Joselina Luzia Menezes Oliveira

**Affiliations:** 1 Brasil Universidade Federal de Sergipe, São Cristóvão, SE – Brasil; 2 Brasil Rede D’Or São Luiz – Clínica e Hospital São Lucas, Aracaju, SE – Brasil; 3 Brasil Rede Primavera – Setor de Métodos Gráficos do Hospital Primavera, Aracaju, SE – Brasil; 4 Brasil Fundação de Beneficência Hospital de Cirurgia – Setor de Métodos Gráficos, Aracaju, SE – Brasil

**Keywords:** Arterial Pressure, Stress Echocardiography, Exercise Test, Coronary Artery Disease, Myocardial Ischemia

## Abstract

**Background:**

The association between exaggerated systolic blood pressure response to exercise (ESBPRE) and myocardial ischemia is controversial and little studied in patients with established or suspected chronic coronary syndrome.

**Objective:**

To verify the relationship between myocardial ischemia and ESBPRE in patients undergoing exercise stress echocardiography (ESE).

**Methods:**

This is a cross-sectional study with 14,367 patients undergoing ESE, from January 2000 to January 2022, divided into the following 2 groups: G1, composed of patients whose peak systolic pressure increased ≥ 90 mmHg (value corresponding to the 95th percentile of the study population), and G2, patients who did not demonstrate an exaggerated hypertensive response. The groups were compared using Student’s t and chi-square tests. P values < 0.05 were considered significant. Logistic regression was also performed to identify independent risk factors for myocardial ischemia, ESBPRE, complaints of typical chest pain prior to the exam, and angina during the test.

**Results:**

Of the 14,367 patients, 1,500 (10.4%) developed ESBPRE, and 7,471 (52.0%) were female. The percentages of previous complaints of typical chest pain, angina during the test, and myocardial ischemia in patients with ESBPRE were 5.8%, 2.4% and 18.1%, compared to 7.4%, 3.9%, and 24.2%, in patients without ESBPRE, respectively (p = 0.021,p = 0.004, p < 0.001). In multivariate analysis, ESBPRE was independently associated with a lower probability of myocardial ischemia (odds ratio: 0.73; 95% confidence interval: 0.58 to 0.93; p = 0.009).

**Conclusion:**

Exaggerated increase in systolic blood pressure during ESE may be a marker for excluding myocardial ischemia.

## Introduction

Exercise stress echocardiography (ESE) is a recognized test for evaluating the functional evolution of coronary disease. ^1 , 2^ This technique provides access to various elements of the ischemic cascade such as angina pectoris, electrocardiographic changes, and changes in segmental contractility and the diastolic function of the left ventricle (LV). ^1 , 3 - 6^

A possible association has been suggested between exaggerated systolic blood pressure response to exercise (ESBPRE) and the presence of myocardial ischemia. ^7 , 8^ It has been speculated that an intense blood pressure elevation during exercise would cause an increase in myocardial oxygen consumption and, consequently, subendocardial ischemia, even in the absence of significant coronary stenoses. ^9 - 11^ Therefore, ESBPRE could be associated with an increased occurrence of cardiovascular events, regardless of cardiorespiratory capacity. ^12^

On the other hand, it has also been speculated that the excessive systolic blood pressure (SBP) response in may be caused by an increase in cardiac output, thus, consisting of a physiological response that translates into favorable prognosis, characterized by a lower probability of chest pain and myocardial ischemia. ^13^ Therefore, the exaggerated increase in SBP during exercise could be a marker of the absence of myocardial ischemia. ^14 , 15^ The decrease in cardiorespiratory capacity would be a determinant of increased mortality. ^16^

The objective of this study was to verify the frequency of myocardial ischemia in patients with suspected or established chronic coronary syndrome (CCS) who did or did not present ESBPRE when undergoing ESE, as well as to compare their clinical and echocardiographic differences.

## Methods

### Patients

This cross-sectional study was based on a prospectively constructed database, which comprised 14,503 patients who underwent ESE between January 2000 and January 2022 at the Laboratório de Ecocardiografia da Clínica e Hospital São Lucas (Echocardiography Laboratory of the São Lucas Clinic and Hospital, abbreviated ECOLAB in Portuguese), a cardiological reference center in Aracaju, Sergipe, Brazil. Patients over 18 years of age referred to the service were included, except those who refused to participate in the study. Patients who had used beta blockers up to 3 days before the exam and those who did not show an increase in SBP above their respective baseline value during physical exercise were also excluded. Therefore, 14,367 patients with suspected or established CCS remained.

Patients were divided according to the presence of ESBPRE, which was defined by an increase of ≥ 90 mmHg (value corresponding to the 95th percentile of the study population). Thus, the following 2 groups were formed: G1, made up of 1,500 (10.4%) patients whose peak SBP increased by ≥ 90 mmHg, and G2, made up of 12,867 (89.6%) patients who did not exhibit an exaggerated hypertensive response.

### Clinical characteristics

Clinical data were collected through interviews carried out before the test. A standardized questionnaire was used to record the following: the occurrence of symptoms such as dyspnea and chest pain, which was considered typical when retrosternal pain triggered by effort or emotional stress and relieved by rest or the use of nitrates and atypical when only 2 of these factors were present. ^17^ The medications used, the presence of risk factors for CCS, and family or personal history of heart disease were also investigated, in addition to data regarding prior coronary syndrome.

Body mass indexes greater than 30 kg/m ^2^ were characterized as obesity. ^18^ Hypercholesterolemia was defined based on personal history and use of lipid-lowering agents (statins and/or fibrates). ^19^ Individuals who reported that they did less than 150 minutes of moderate-intensity physical activity or less than 75 minutes of vigorous-intensity activity were considered sedentary. ^20^ Systemic arterial hypertension (SAH) was considered when blood pressure levels measured in the upper limb, at rest and in ideal conditions, were SBP ≥ 140 mmHg and/or diastolic blood pressure ≥ 90 mmHg, repeated and confirmed, or when the patient was using antihypertensive medication. ^21^

Diabetes mellitus was defined by the presence of fasting blood glucose ≥ 126 mg/dL, blood glucose 2 hours after a 75 g glucose overload ≥ 200 mg/dL, glycated hemoglobin ≥ 6.5%, or random blood glucose ≥ 200 mg/dL associated with classic symptoms of hyperglycemia, or the use of insulin or oral hypoglycemic agents. ^22^

Old myocardial infarction was defined based on clinical history and/or the presence of suggestive changes in previous complementary tests, such as electrocardiogram (ECG), echocardiogram, and/or coronary cineangiography. ^6^

The isolated or combined indications for ESE were as follows: assessment of chest pain; preoperative assessment for non-cardiac surgery; positive exercise test for myocardial ischemia in patients with low risk of coronary syndrome; positive exercise test in patients with intermediate or high risk of coronary syndrome; negative exercise test for myocardial ischemia in patients with intermediate or high risk of coronary syndrome; appearance of arrhythmia during exercise test; stratification of previously established CCS; and risk stratification after acute coronary syndrome. ^3 , 23^ Exercise tests were considered suggestive of ischemia when they presented ST segment depressions during the exercise or recovery phase with any of the following: horizontal or descending morphology > 1 mm, measured at the J point; ascending morphology > 1.5 mm, in patients with moderate or high risk of coronary syndrome; and > 2 mm in patients with low risk of coronary syndrome, measured at the Y point. ^24^ The risk classifications for coronary syndrome followed the recommendations by Knuuti et al. ^5^ Although ECG tracings at rest with the presence of left bundle branch block or changes in the ST segment represent limitations ^25^ to the aforementioned ECG criteria, in these cases, the other segments that did not involve these findings were analyzed, given the applicability of ESE as a predictor of coronary syndrome in these scenarios. ^2 , 26^

### Exercise stress echocardiography

The basic experimental protocol consisted of a 12-lead ECG and resting echocardiogram after clinical evaluation. Subsequently, physical exercise was performed on a treadmill, and, immediately (30 seconds to 1 minute) afterwards, echocardiographic images were acquired in the immediate post-exercise period for a period of 2 to 5 minutes using simultaneous side-by-side analysis of the images obtained at rest for comparative evaluation of LV segmental contractility, as recommended by the American Society of Echocardiography. ^2^ All patients underwent the standard Bruce or Ellestad protocol during the exercise test. ^24^ All patients were encouraged to reach maximum peak heart rate (HR), estimated by the equation: 220 – age; submaximal HR was defined as 85% of maximum HR. ^24^ Reasons for interrupting exercise before reaching maximum HR included the development of angina, syncope, fatigue, and malignant arrhythmias, or at the patient’s discretion due to muscle fatigue. Oxygen consumption at peak exercise (VO _2_ max) was obtained indirectly, through standardized metabolic calculations that estimated aerobic capacity at each stage of the aforementioned protocols. ^27^ The VO _2_ max predicted according to sex, age, body mass index, and level of physical activity was determined based on the equations by Almeida et al. ^28^ for the Brazilian population. The load was also expressed in metabolic equivalents (MET), where 1 MET corresponds to 3.5 mL/kg·min of inhaled VO _2_ at rest. ^29^ During exercise, patients were continuously monitored with ECG. The occurrence of ST segment depressions that met the same criteria mentioned above for exercise tests suggestive of myocardial ischemia were considered ischemic ECG changes during exercise. ^24^

Patients were observed before, during, and after physical exercise, using a 12-lead ECG, to check for possible complications arising from exercise stress, which were classified according to the definitions by Geleijnse et al. ^30^ The presence of death, acute myocardial infarction, stroke, cardiac rupture, ventricular fibrillation, and cardiac asystole were considered major complications. Minor complications were defined as atrioventricular block, coronary spasm, ventricular arrhythmias (non-sustained ventricular tachycardia and ventricular extrasystoles), and supraventricular arrhythmias (atrial fibrillation or flutter, non-sustained supraventricular tachycardia, and supraventricular extrasystoles). ^30^

Suspension of negative chronotropic drugs, such as beta blockers, was recommended at least 3 days before the tests, while maintaining the patient’s other usual medications. ^2^

The tests were performed with Hewlett Packard/Phillips SONOS 5500 equipment until 2012 and, subsequently, with a Phillips IE-33 echocardiography device, observing the effective technical aspects described by the American Society of Echocardiography. ^31 - 33^ The 2-dimensional echocardiographic images were obtained in the parasternal and apical windows, during rest and immediately after exercise, with the patient in the left lateral decubitus position and under simultaneous ECG recording. The segmental LV wall motion was evaluated by an experienced echocardiographer, as recommended by the American Society of Echocardiography. ^2^ Segmental LV wall thickening was evaluated quantitatively both at rest and after exercise, using the 16-segment methodology, graded as follows: 1, normal; 2, hypokinetic; 3, akinetic; and 4, dyskinetic. The left ventricular wall motion score index (LVWMSI) was calculated at rest and during exercise as the sum of the scores assigned to each of the 16 segments divided by the number of segments evaluated at the given moment. An LVWMSI equal to 1 corresponds to normality; 1.1 to 1.7 represents intermediate dysfunction; and greater than 1.7 represents significant dysfunction. ^2^ The difference between the LVWMSI during exercise and at rest is known as Δ LVWMSI. The development of a new change in wall motion or worsening of existing dyssynergy (ΔLVWMSI ≠ 0) was considered indicative of myocardial ischemia. ^31 - 33^ LV diastolic function was evaluated and classified according to the current recommendations of the American Society of Echocardiography. ^34 , 35^

### Statistical analysis

Quantitative variables were described as mean and standard deviation. According to the assumption of sample normality for all variables, as assessed by the Kolmogorov-Smirnov test, they were analyzed using Student’s t test for independent groups. Categorical variables were displayed as absolute frequency and percentage. To compare the characteristics of the categorical variables between both groups, the chi-square test was used. A significance level of 5% was adopted for all analyses. To evaluate the association between the outcomes (ESBPRE, myocardial ischemia and

angina on ESE, and previous complaint of typical chest pain) and the associated factors, logistic regressions were performed using the hierarchical method. To be inserted into the initial model, all variables with p < 0.25 were admitted, whereas, to remain in the multivariate analysis model, p < 0.05 was adopted. The variables were added and removed from the model manually, according to the aforementioned criteria. Statistical analyses were processed using the Statistical Package for the Social Sciences (SPSS), version 22.0 (Chicago, IL, USA). ^36^

### Ethical aspects

The ethical principles governing experimentation in humans were carefully followed, and all patients signed a free and informed consent form. The study received approval from the Research Ethics Committee of the Federal University of Sergipe (CAAE 1818.0.000.107-06).

## Results

### Baseline characteristics

The sample consisted of 14,367 patients, with a mean age of 58 ± 11 years, 52.0% of whom were women. A total of 1,500 (10.4%) patients developed ESBPRE ( Table 1 ). The proportion of men in the group who demonstrated an exaggerated increase in SBP was greater than in the group that did not develop ESBPRE. On average, individuals with ESBPRE were younger than those without excessive response. The main clinical indication for ESE was the assessment of chest pain, present in 52.6% of individuals. In some cases, there was more than one indication for the exam.

**Table 1:** – Clinical characteristics of patients who did or did not present exaggerated systolic blood pressure response to exercise (ESBPRE)

Variables	ESBPRE n=1500 (10.4%)	Without ESBPRE n=12,867 (89.6%)	p ^*^
Male sex	1008(67.2%)	5888(45.8%)	<0.001
Age (mean, years)	54.6±10.3	58.1±11.4	<0.001
Systemic arterial hypertension	972(65.5%)	7459(58.7%)	<0.001
Diabetes mellitus	202(13.6%)	1651(13.0%)	0.516
Dyslipidemia	819(55.2%)	7353(57.9%)	0.041
Smoking	88(5.9%)	631(5.0%)	0.110
Sedentary lifestyle	602(53.2%)	4783(52.7%)	0.726
Obesity	489(32.8%)	2859(22.3%)	<0.001
Family history of CCS	831(56.0%)	7283(57.4%)	0.323
Personal history of CCS	188(19.7%)	1738(21.4%)	0.242
Clinical treatment for CCS ^†^	83(44.1%)	795(45.7%)	0.677
Angioplasty + stent ^†^	78(41.5%)	680(39.1%)	0.529
Surgical revascularization ^†^	32(17.5%)	344(20.1%)	0.397
Old infarction (> 60 days)	76(5.3%)	680(5.5%)	0.730
Recent infarction (< 60 days)	0(0.0%)	48(0.4%)	0.018
**Symptoms ^**‡**^**			
– Asymptomatic	648(45.0%)	5209(42.0%)	0.028
– Typical chest pain prior to the test	83(5.8%)	922(7.4%)	0.021
– Atypical chest pain prior to the test	644(44.7%)	5630(45.4%)	0.642
– Dyspnea prior to the test	69(4.8%)	731(5.9%)	0.091
Left bundle branch block	42(2.8%)	534(4.2%)	0.012
**Use of antihypertensive medication**	723(49.4%)	6020(47.8%)	0.271
– Diuretics	17(3.7%)	200(4.1%)	0.657
– ACEI	159(10.9%)	1159(9.2%)	0.040
– ARB	346(23.7%)	2672(21.3%)	0.034
– Calcium channel blocker	121(8.3%)	918(7.3%)	0.178
– Beta blocker	303(20.7%)	2887(23.0%)	0.052
– Nitrate	40(2.7%)	357(2.8%)	0.814
**Clinical indications for ESE ^**§**^**			
– Investigation of chest pain	727(50.5%)	6552(52.8%)	0.097
– Preoperative assessment for non-cardiac surgery	58(6.2%)	585(7.4%)	0.170
– SET in patients with low risk of coronary syndromes	221(23.6%)	1675(21.2%)	0.099
– SET in patients with intermediate or high risk of coronary syndromes	41(4.4%)	436(5.6%)	0.133
– NET in patients with intermediate or high risk of coronary syndromes	12(1.3%)	112(1.4%)	0.730
– Arrhythmias during ET	1(0.1%)	48(0.6%)	0.051
– CCS risk stratification	188(19.7%)	1738(21.4%)	0.242

ACEI: angiotensin-converting enzyme inhibitor; ARB: angiotensin II receptor blocker; CCS: chronic coronary syndrome; ESE: exercise stress echocardiography; ET: exercise test; NET: negative exercise test for myocardial ischemia; SET: exercise test suggestive of myocardial ischemia. ( ^*^ ) Qualitative variables were calculated using Pearson’s chi-square method, and quantitative variables were calculated using Student’s t test for independent samples, in accordance with the assumption of sample normality. ( ^†^ ) For the analysis of CCS therapies (clinical treatment, angioplasty + stent, and surgical revascularization), only patients with coronary artery disease were considered (n = 1926). ( ^‡^ ) For the analysis of symptoms, only complete data were considered (n = 13,936). ( ^§^ ) For the analysis of clinical indications, only complete data were considered (n = 12,394). Source: Data collected by the authors.

Patients with ESBPRE had a lower frequency of dyslipidemia and a greater frequency of SAH and obesity. Furthermore, the group with ESBPRE used more angiotensin-converting enzyme inhibitors and angiotensin II receptor blockers. Regarding symptoms, the majority of asymptomatic patients belonged in the ESBPRE group, while individuals without exaggerated systolic increase demonstrated a higher frequency of previous complaints of typical chest pain than those with ESBPRE ( Table 1 ).

### Exercise stress echocardiography

There were no records of major complications. The majority of patients who reported angina during the test were in the group without ESBPRE ( Table 2 ). There was a higher frequency of ST depression in the group with ESBPRE. In patients without ESBPRE, there was a higher frequency of echocardiographic changes compatible with the presence of myocardial ischemia ( Figure 1 ), as well as higher LVWMSI levels at rest and during exercise ( Table 2 ).

**Table 2:** – Echocardiographic and exercise characteristics of patients who did or did not present exaggerated systolic blood pressure response to exercise (ESBPRE)

Variables	ESBPRE n=1500 (10.4%)	Without ESBPRE n=12,867 (89.6%)	p ^*^
**Results of segmental motion ^**†**^**			
– Normal	1227(81.9%)	9751(75.8%)	<0.001
– Induced ischemia	127(8.5%)	1563(12.2%)	<0.001
– Fixed ischemia	121(8.1%)	1151(9.0%)	<0.001
– Fixed and induced ischemia	24(1.6%)	391(3.0%)	0.002
Resting SBP (mmHg)	123.6±12.1	128.6±12.7	<0.001
Peak SBP (mmHg)	219.3±16.3	189.1±16.6	<0.001
SBP after exercise (mmHg)	156.5±25.8	145.0±20.7	<0.001
Resting HR (bpm)	76.9±13.1	77.9±14.0	0.005
Peak HR (bpm)	159.8±16.8	154.1±19.2	<0.001
HR after exercise (bpm)	101.7±15.3	98.0±18.3	<0.001
Resting DP (×103 mmHg.bpm)	9.5±2.0	10.0±2.2	<0.001
Peak DP (×103 mmHg.bpm)	35.0±4.0	29.2±4.7	<0.001
DP after exercise (×103 mmHg.bpm)	15.9±3.7	14.2±3.5	<0.001
% of theoretical maximum HR	96.7±8.3	95.2±9.7	<0.001
**HR reached on the test ^**‡**^**			
– Below submaximal	119(7.9%)	1657(12.9%)	<0.001
– Submaximal	619(41.3%)	5138(40.0%)	0.329
– Maximum	211(14.1%)	1392(10.8%)	<0.001
– Above maximum	551(36.7%)	4670(36.3%)	0.754
Angina during the test	36(2.4%)	500(3.9%)	0.004
Dyspnea during the test	164(11.0%)	1421(11.1%)	0.951
**ST depression**	883(59.3%)	6824(53.8%)	<0.001
– Ascending ^§^	325(36.8%)	2661(39.0%)	0.209
– Horizontal ^§^	318(36.0%)	2669(39.1%)	0.075
– Descending ^§^	241(27.3%)	1499(22.0%)	<0.001
Atrioventricular block	3(0.9%)	18(0.5%)	0.304
Coronary spasm	0(0.0%)	1(0.0%)	0.766
Non-sustained VT	1(0.3%)	24(0.6%)	0.448
Ventricular extrasystole	75(21.6%)	1012(25.8%)	0.088
Atrial fibrillation or flutter	0(0.0%)	8(0.2%)	0.399
Non-sustained SVT	5(1.4%)	59(1.5%)	0.925
Supraventricular extrasystole	30(8.7%)	406(10.4%)	0.313
Treadmill time (minutes)	8.2±2.6	7.1±2.6	<0.001
Functional capacity (METs)	10.5±3.1	9.7±3.0	<0.001
Predicted VO _2_ max (mL.kg ^-1^ .min ^-1^ )	26.3±6.0	24.5±6.2	<0.001
VO _2_ max achieved (mL.kg ^-1^ .min ^-1^ )	36.7±10.7	34.1±10.5	<0.001
Ratio between predicted and achieved VO _2_ max (%)	143.2±38.9	143.2±42.8	0.986
LV ejection fraction (%)	67.1±6.4	67.1±6.7	0.808
LV mass index (g/m ^2^ )	88.6±22.1	84.9±22.9	<0.001
Left atrial volume (mL/m ^2^ )	28.8±13.1	29.3±14.2	0.400
Resting LVWMSI	1.02±0.1	1.03±0.1	0.001
LVWMSI during exercise	1.03±0.1	1.04±0.1	<0.001
**Diastolic function ^**//**^**			
– Normal	230(20.4%)	1998(22.2%)	0.163
– Grade I dysfunction	618(54.7%)	5016(55.7%)	0.531
– Grade II dysfunction	277(24.5%)	1950(21.7%)	0.028
– Grade III dysfunction	4(0.4%)	38(0.4%)	0.738

DP: double product; HR: heart rate; LV: left ventricle; LVWMSI: left ventricular wall motion score index; SBP: systolic blood pressure; SVT: supraventricular tachycardia; VO _2_ max: peak oxygen consumption; VT: ventricular tachycardia. ( ^*^ ) Qualitative variables were calculated using Pearson’s chi-square method, and quantitative variables were calculated using Student’s t test for independent samples, in accordance with the assumption of sample normality. ( ^†^ ) For the analysis of segmental motion, only complete data were considered (n = 14,355). ( ^‡^ ) For analysis of HR achieved, only complete data were considered (n = 14,357). ( ^§^ ) For analysis of the morphologies of ST segment depression (ascending, horizontal, and descending), only patients who presented ST segment depression were considered (n = 7707). ( ^//^ ) For analysis of diastolic function, only complete data were considered (n = 10,131). Source: Data collected by the authors.

**Figure 1:** – Comparison of the frequency of myocardial ischemia on exercise stress echocardiography between groups of patients who did or did not present exaggerated systolic blood pressure response to exercise (ESBPRE). Source: Data collected by the authors.

In the multivariate analysis, a significant inverse relationship was maintained between myocardial ischemia and ESBPRE ( Table 3 ). The biggest predictor of myocardial ischemia was the appearance of angina during the test ( Table 4 ). ESBPRE was not associated with typical chest pain prior to the test ( Table 5 ), nor with the appearance of angina during the test ( Table 6 ).

**Table 3:** – Multivariate logistic regression with parameters associated with exaggerated systolic blood pressure response to exercise on stress echocardiography

Variables	Odds ratio	95% CI	p
Myocardial ischemia	0.72	0.59-0.87	0.001
Male sex	2.38	2.05-2.77	<0.001
Age	0.98	0.97-0.99	<0.001
Systemic arterial hypertension	1.42	1.21-1.65	<0.001
Chronic coronary syndrome	0.85	0.70-1.03	0.103
Obesity	1.54	1.32-1.79	<0.001
Typical angina prior to the test	0.83	0.61-1.13	0.229
Typical pain during the test	0.81	0.52-1.27	0.367

CI: confidence interval. Source: Data collected by the authors.

**Table 4:** – Multivariate logistic regression with parameters associated with the presence of myocardial ischemia on exercise stress echocardiography

Variables	Odds ratio	95% CI	p
ESBPRE	0.73	0.58-0.93	0.009
Male sex	1.33	1.15-1.55	<0.001
Age	1.00	1.00-1.01	0.361
Systemic arterial hypertension	1.10	0.94-1.28	0.241
Diabetes mellitus	1.32	1.10-1.58	0.003
Dyslipidemia	1.39	1.21-1.61	<0.001
CCS	3.69	3.16-4.30	<0.001
Tobacco use	1.44	1.08-1.91	0.012
Family history of CCS	1.38	1.19-1.59	<0.001
Typical angina prior to the test	2.38	1.87-3.02	<0.001
Angina during the test	11.03	7.60-16.01	<0.001
ST depression	1.53	1.33-1.76	<0.001
Functional capacity (METs)	0.94	0.91-0.97	<0.001
LV ejection fraction (%)	0.94	0.93-0.95	<0.001

CCS: chronic coronary syndrome; CI: confidence interval; ESBPRE: exaggerated systolic blood pressure response to exercise; LV: left ventricle. Source: Data collected by the authors.

**Table 5:** – Multivariate logistic regression with parameters associated with the presence of complaints of typical chest pain prior to the test

Variables	Odds ratio	95% CI	p
ESBPRE	0.80	0.56-1.14	0.218
Echocardiographic myocardial ischemia	2.37	1.88-3.00	<0.001
Male sex	1.05	0.84-1.31	0.676
Age	0.99	0.98-0.995	0.003
Systemic arterial hypertension	1.06	0.85-1.33	0.602
Diabetes mellitus	1.10	0.84-1.44	0.501
Dyslipidemia	1.32	1.06-1.64	0.013
Chronic coronary syndrome	1.01	0.78-1.30	0.964
Tobacco use	1.81	1.24-2.64	0.002
Angina during the test	6.52	4.73-8.99	<0.001
ST depression	0.89	0.72-1.09	0.256
Functional capacity (METs)	0.95	0.91-0.99	0.008

CI: confidence interval; ESBPRE: exaggerated systolic blood pressure response to exercise. Source: Data collected by the authors.

**Table 6:** – Multivariate logistic regression with parameters associated with the appearance of angina during exercise stress echocardiography

Variables	Odds ratio	95% CI	p
ESBPRE	1.12	0.68-1.85	0.651
Myocardial ischemia	10.37	7.21-14.91	<0.001
Male sex	1.63	1.17-2.26	0.004
Age	0.98	0.96-0.99	0.003
Systemic arterial hypertension	1.01	0.72-1.42	0.950
Diabetes mellitus	1.04	0.71-1.51	0.856
Dyslipidemia	0.89	0.65-1.22	0.471
Chronic coronary syndrome	1.02	0.73-1.43	0.892
Typical angina prior to the test	6.51	4.72-8.97	<0.001
ST depression	1.70	1.25-2.32	0.001
Functional capacity (METs)	0.83	0.78-0.88	<0.001

CI: confidence interval; ESBPRE: exaggerated systolic blood pressure response to exercise. Source: Data collected by the authors.

## Discussion

In this study, ESBPRE was associated with a lower probability of complaint of prior typical chest pain, exercise-induced angina, and myocardial ischemia on ESE, with lower LVWMSI during rest and exercise. Nonetheless, the group that presented ESBPRE was also associated with a greater presence of ECG changes suggestive of myocardial ischemia.

The association between ST segment depression and ESBPRE observed in this study can be explained by the relationship between the systolic increase and false positives for myocardial ischemia on ECG, described in previous studies. ^37 , 38^ Another possible theory would be the presence of coronary microvascular disease in patients with ST depression, ^39^ which would cause distinct functioning of the heart pump compared to individuals with obstructive coronary syndrome and would not prevent the first group from reaching higher blood pressure levels. Daubert et al. ^40^ observed that the combination of an ECG suggestive of myocardial ischemia with a normal exercise echocardiogram was associated with higher blood pressure levels at peak ESE.

Unlike other studies, our sample was mostly composed of women (52.0%); nevertheless, the predilection of ESBPRE for the male sex was maintained. This finding has also been verified in the literature, in addition to the predominance of younger patients with exaggerated systolic response to exercise. ^41 - 44^ No major complications were recorded in the present study. ESE is a modality with a low prevalence of adverse events that represent a risk of death. ^45^

This study showed that patients with ESBPRE achieved greater MET values. This situation is possibly linked to the fact that the ESBPRE group included younger individuals, and it was, consequently, easier to increase the double product, increasing cardiac work. ^42 , 44 , 46^ Kokkinos et al. ^16^ found an association between low cardiorespiratory capacity and increased all-cause mortality, and the risk was substantially higher (47% versus 92%) among those who did not reach an increase above 52 mmHg in SBP during peak exercise. This finding corroborates the concept that the SBP response to exercise provides essential information about the integrity of the cardiovascular system. ^13^

The predominance of SAH in the ESBPRE group was expected, as SAH makes it possible to reach higher blood pressure levels, a pattern also observed in other studies. ^14 , 41 , 44^ As for other cardiovascular risk factors, patients with an exaggerated systolic increase were also associated with obesity in the present study. Giang et al. ^46^ demonstrated that patients with high blood pressure levels during exercise had higher body mass indexes. Furthermore, dyslipidemia was more prevalent among patients without ESBPRE in our sample. Bouzas-Mosquera et al. ^41^ found a distinct association, in which dyslipidemia was correlated with ESBPRE.

There are reports that the use of antihypertensive medication does not have a significant influence on ESBPRE. ^14 , 41 - 44^ On the contrary, our sample showed an association between ESBPRE and greater use of angiotensin-converting enzyme inhibitors and angiotensin II receptor blockers. However, this finding can be justified by the fact that the majority of patients with hypertension were in this group, and both medications compose the first line in the treatment of SAH. ^21^

Cardiorespiratory capacity and the individual’s previous history should be considered during assessment of ESBPRE, especially in relation to athletes. In a population of athletes, Caselli et al. ^47^ found cutoff values for ESBPRE above 220 mmHg for men and 200 mmHg for women, and they also demonstrated an association between exaggerated systolic increase and endurance sports (defined as primarily isotonic activities) and modalities with isotonic and isometric components. Accordingly, athletes from these specific sports could present ESBPRE without, however, being associated with pathologies such as SAH. ^47^

We opted to define ESBPRE as an increase in SBP greater than or equal to the 95th percentile of our population in order to adapt the increase to the intrinsic characteristics of the sample, a parameter also used in other studies, ^42 - 44^ allowing reproducibility. In these studies, carried out in a European population, the deltas in systolic increase referring to the 95th percentile of their respective samples were values lower than those of our population (≥ 80 and ≥ 70 mmHg). ^42 - 44^

Our results do not invalidate the beneficial effect that arises from reducing both systolic pressures (at rest and peak effort) induced by regular physical activity. ^48^ In our sample, the mean value of the LV mass index was higher among individuals with ESBPRE, which may be due to LV remodeling induced by high blood pressure levels. Perçuku et al., ^8^ based on a meta-analysis of 8 longitudinal studies that included a total of 47,188 patients without coronary artery disease, concluded that ESBPRE constitutes an independent risk factor for cardiovascular events and mortality. Future analyses are necessary to verify whether the inverse relationship between myocardial ischemia and ESBPRE found in the present study could indicate an independent protective factor for cardiovascular events.

There was no association between positive personal history for CCS and the absence of ESBPRE in our study, a situation also observed in European studies. ^42 , 43^ Nonetheless, in the present study, ESBPRE remained a protective factor against the appearance of myocardial ischemia in multivariate analysis. Another study with angiography data demonstrated a lower frequency of coronary disease in patients with ESBPRE. ^13^

During physical stress, a progressive SBP increase is expected in a healthy individual, concomitantly with an increase in cardiac work, while diastolic blood pressure remains constant or slightly reduced. ^49^ The increase in SBP results from an increase in cardiac output, which, in turn, originates from increases in HR and stroke volume. Simultaneously, the sympathetic response redistributes blood flow to areas with greater metabolic need, producing muscular vasodilation and vasoconstriction in inactive areas, which explains the slight reduction in diastolic blood pressure. ^49 , 50^ Although an increase in cardiac output provides better prognoses, an unexpected increase in peripheral vascular resistance in circumstances of physical stress may be an indicator of worse prognosis. ^49^

These differences between both determinants of increased SBP during exercise (increased cardiac output and increased peripheral vascular resistance) may, in part, explain the inconsistencies present between different studies regarding the presence of ESBPRE and the effect on all-cause mortality. Accordingly, Daubert et al. ^40^ observed that individuals with myocardial ischemia on ESE achieved lower double product. In our study, individuals with ESBPRE started with lower SBP values and showed a greater increase in both SBP and HR with exercise, which corroborates the hypothesis that the increase in cardiac output may be the main determinant for the present results.

### Limitations

Our study has the limitations that are inherent to cross-sectional observational studies whose sample comes from a single center; therefore, we highlight that understanding the influence of ESBPRE on mortality and cardiovascular events requires longitudinal studies that can observe outcomes in this population. It is worth underscoring the fact that the results refer to patients with established or suspected CCS. Furthermore, despite the exclusion of patients treated with beta blockers up to 3 days before the test, we cannot rule out a residual effect of these drugs. We reiterate that patients who did not show an increase in SBP above the baseline value during exercise were excluded.

## Conclusions

ESBPRE during ESE may be a marker associated with the absence of myocardial ischemia in patients with known or suspected CCS. Therefore, the use of ESE represents an important advantage with respect to the possibility of accessing the patient’s aerobic capacity and, therefore, understanding the behavior of the SBP response to exercise. Furthermore, it is worth underscoring that the prognostic value of exaggerated systolic increase will require further studies.
